# Estimating parameters of a stochastic cell invasion model with fluorescent cell cycle labelling using approximate Bayesian computation

**DOI:** 10.1098/rsif.2021.0362

**Published:** 2021-09-22

**Authors:** Michael J. Carr, Matthew J. Simpson, Christopher Drovandi

**Affiliations:** School of Mathematical Sciences, Queensland University of Technology, Brisbane, Australia

**Keywords:** sequential Monte Carlo, SMC-ABC, cell proliferation, cell motility, random walk model

## Abstract

We develop a parameter estimation method based on approximate Bayesian computation (ABC) for a stochastic cell invasion model using fluorescent cell cycle labelling with proliferation, migration and crowding effects. Previously, inference has been performed on a deterministic version of the model fitted to cell density data, and not all parameters were identifiable. Considering the stochastic model allows us to harness more features of experimental data, including cell trajectories and cell count data, which we show overcomes the parameter identifiability problem. We demonstrate that, while difficult to collect, cell trajectory data can provide more information about the parameters of the cell invasion model. To handle the intractability of the likelihood function of the stochastic model, we use an efficient ABC algorithm based on sequential Monte Carlo. Rcpp and MATLAB implementations of the simulation model and ABC algorithm used in this study are available at https://github.com/michaelcarr-stats/FUCCI.

## 1. Introduction

Australia and New Zealand have the highest incidence rates of melanoma in the world, followed by northern America and northern Europe [1]. In Australia, melanoma is the third most common diagnosed form of cancer [2]. Since the 1960s, Australia’s primary strategy to reduce overall mortality rates has been targeted at early prevention and detection [3]. However, a better understanding of the mechanisms which control cell invasion is necessary in order to improve or establish new treatment measures.

The underlying mechanisms of cell invasion we consider are combined cell proliferation and cell migration. Cell proliferation is a four-stage sequence consisting of gap 1 (G1), synthesis (S), gap 2 (G2) and mitosis (M) where the cell divides into two daughter cells, each of which return to the G1 phase [4]. Improvements in technology have enabled us to visualize different phases of the cell cycle in real time using fluorescent ubiquitination-based cell cycle indicator (FUCCI) technology [5]. FUCCI technology involves two fluorescent probes which emit red fluorescence when the cells are in G1 phase and green fluorescence when in S/G2/M phases. During the transition between G1 and S phase, both probes are active (giving the impression that the cell fluoresces yellow), allowing the visualization of the early S phase, which we refer to as eS. Experiments using FUCCI-transduced melonama cells are becoming increasingly important in cancer research because many drug treatments target different phases of the cell cycle [6].

The development of simulation models offer us a quick and inexpensive alternative to *in vitro* experiments. Although, existing mathematical models have had a long history without incorporating cell cycle information until more recently (e.g. [7,8]). In this study, we adopt the cell invasion model of scratch assay experiments developed by Simpson *et al.* [7]. This model describes a discrete exclusion based random walk on a two-dimensional (2D) hexagonal lattice. Furthermore, this model involves treating the entire population of agents as three subpopulations that correspond to the red, yellow and green phases of the cell cycle as identified by FUCCI. Agents transition through the cell cycle, while simultaneously undergoing a nearest neighbour random walk, with exclusion, to model cell migration. This model is discussed in more detail in §3.1. This previous study did not perform any parameter inference or calibrate the model to experimental data. The primary focus of this present work is to apply Bayesian methods to recover parameter estimates for the model and the associated distribution of uncertainly around them. However, standard Bayesian approaches rely on the computation of the likelihood function which is often intractable in complex stochastic models. We overcome this limitation by applying approximate Bayesian computation (ABC) methods, which is discussed later in §3.2.

Simpson *et al.* [9] investigate practical parameter identifiability in a deterministic partial differential equation of FUCCI scratch assay experiments. Practical parameter identifiability is a term that describes whether it is possible to produce precise estimates with finite regions of confidence levels [10]. We adopt this terminology here since it is consistent with Simpson *et al.* [9]. Nevertheless, by using a simpler model, their study was able to adopt standard Bayesian approaches to parameter estimation since the likelihood function is tractable. Using a Markov chain Monte Carlo (MCMC) framework and cell density data, their study found cell diffusivities were practically non-identifiable when they considered the case where the cell migration rate depends on the cell cycle phase. Although, their study does not consider other types of data which may be more informative of the underlying mechanisms. Here, we address the limitations Simpson *et al.* [9] identify by modelling individual cell behaviour with a stochastic model which allows the generation of numerous data types. Indeed, we take full advantage of the flexibility of the stochastic model in this study and combine multiple data types (the number of cells in each phase and cell trajectory data accounting for different phases) to improve parameter identifiability. However, working with cell trajectory data can be challenging, and these challenges include time consuming effort to manually track cells and the need for the cell density to be low to make cell tracking easier. Models that can avoid using cell trajectory data are an active area of research [11], but we find using the Simpson *et al.* [7] model, which incorporates cell trajectory data, leads to a good outcome.

Many other studies have explored modelling and/or parameter estimation in cell invasion models [12–17]. Notably, Vo *et al.* [12] estimate the parameters of a stochastic cell spreading model of an expanding population of fibroblast cells in a 2D circular barrier assay without cell cycle labelling. While ABC methods have previously been considered in stochastic cell spreading models, such as the Vo *et al.* [12] study, they have never before been considered with FUCCI models and/or data. Prior to Vo *et al.* [12], cell invasion models were usually defined by deterministic partial differential equation and when performing parameter inference, they usually used trial and error-based approaches [16] or non-linear least-squares estimation [13–15,17]. However, these approaches to parameter estimation are unable to quantify the uncertainty around the point estimates. In this study, we show that using a discrete stochastic model is necessary to identify the transition and motility parameters when multiple phases of the cell cycle are considered. This difference is due to the wider range of data types that are available since individual cells are modelled rather than working with a simple cell density profile. This allows data types that are more informative about the model parameters, which have previously been unavailable to deterministic modelling approaches, to be considered. That is, we find cell count and cell trajectory data to be the most informative data types as they can produce practically identifiable parameters for the transition and motility parameters, respectively. Rcpp and MATLAB implementations of the simulation model and ABC algorithm used in this study are available at https://github.com/michaelcarr-stats/FUCCI.

The paper is structured as follows. In §2, we introduce the experimental data and the process by which it is collected. §3 describes the simulation model, the parameter inference method used, and our prior knowledge on the model parameters. In §4, we explain the image analysis process and present the inference results when using synthetic and experimental data sets. Discussion of results, future work and concluding remarks are presented in §5.

## 2. Data

**Figure 1.**
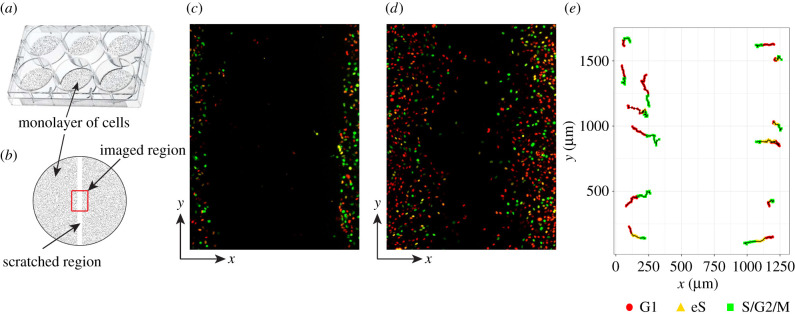
Experimental procedure and data. (*a*,*b*) Explains the experimental procedure and boundary conditions for simulation models. (*a*) Photograph of six culture plates commonly used with a uniform monolayer of cells. (*b*) Schematic showing the uniform cell monolayer (shaded), scratched region (white) and imaged region (outlined in red) in a 35 mm culture plate. (*c*,*d*) Experimental images, both 1309.09 × 1745.35 μm, of WM983C FUCCI-transduced melanoma cells at 0 and 48 h, respectively. Images reproduced with permission from Vittadello *et al.* [20]. (*e*) Cell trajectory data of a select few cells recorded through red to green phases travelling inward to fill scratched region.

2D scratch assay experiments are a good screening tool for more complex experimental models, as they are low cost, allow for easy data interpretation and readily allow control of oxygen, nutrients and drug supply [18,19]. We adopt data from a study conducted by Vittadello *et al.* [20] where a scratch assay is used to examine melanoma cell proliferation and migration in real time with FUCCI technology. The experiment is initialized by placing a small population of cells and a growth medium in a culture dish (figure 1*a*) to create a uniform 2D monolayer of cells. Next, a sharp-tipped instrument is used to make a scratch in the monolayer of cells (figure 1*b*). Finally, the cells are observed at regular intervals as they proliferate and migrate into the newly created gap over the following 48 h. For this study, we adopt the data from the experiments with WM983C FUCCI-transduced melonoma cells and present still images captured at 0 and 48 h in figure 1*c*,*d*, respectively. A major advantage of 2D scratch assay experiments is the multitude of different data types which can be easily recovered. The data types which we explore later include the number of cells in each population, position of cell populations, and cell trajectory data (figure 1*e*). It is important to consider the size of the imaged region compared to the culture plate (figure 1*b*) because the boundaries of the imaged region are not physical boundaries. Since the cell density outside of the scratched region is approximately uniform, with no macroscopic density gradients away from the leading edge, the net flux of cells across the boundary will be zero [7]. Therefore, the appropriate mathematical boundary conditions along the vertical boundaries will be zero net flux.

## 3. Methods

### 3.1. Simulation model

We adopt the discrete random walk model developed by Simpson *et al.* [7] on a 2D hexagonal lattice. Each lattice site has diameter Δ = 20 μm, which is the average cell diameter [21], and is associated with a set of unique Cartesian coordinates,

3.1 $$(x_{i},y_{j})=\left\{ \begin{array}{ll} \left((j-1/2)\Delta \sqrt{3}/2,i\Delta \right) & \mathrm{if}\;i\;\mathrm{is}\;\mathrm{even},\\ \left((j-1)\Delta \sqrt{3}/2,i\Delta \right)\; & \mathrm{if}\;i\;\mathrm{is}\;\mathrm{odd},\; \end{array}\right.$$

where *i* and *j* are the respective row and column indices. To mimic scratch assay experiments, cells in G1 phase are represented by red agents, cells in eS phase are represented by yellow agents, and cells in S/G2/M phase are represented by green agents. Agents are permitted to transition through phases of the cell cycle and undergo a nearest neighbour random walk by simulating from a Markov process using the Gillespie algorithm [22] where the time between events is exponentially distributed. The algorithm is presented in §1 of the electronic supplementary material.

**Figure 2.**
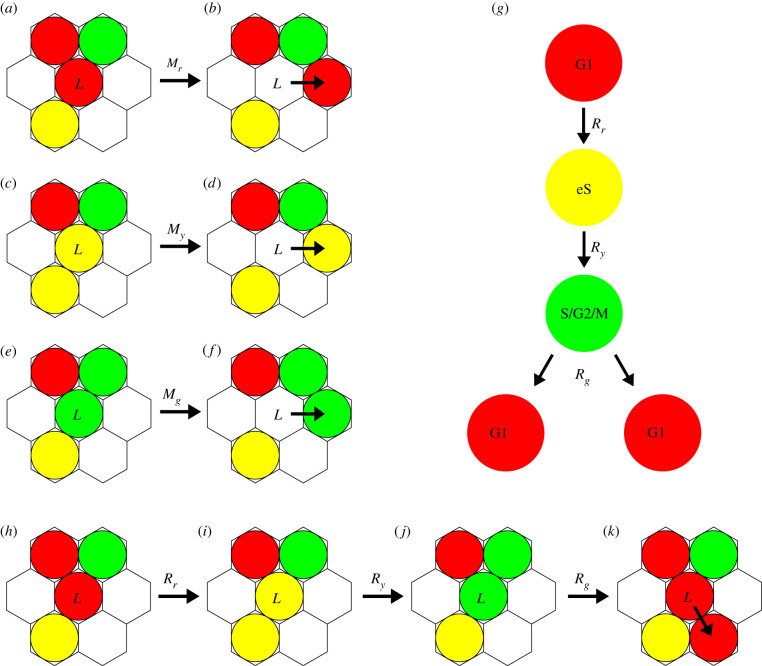
Cell migration and proliferation. (*a*–*f*) An agent at lattice site *L* will attempt to migrate to the six neighbouring lattice sites, successfully migrating if the selected site is vacant. (*g*) Schematic showing the progression through the G1 phase (red), early S phase (yellow) and S/G2/M phase (green) for FUCCI. (*h*–*k*) Agent transition through the cell cycle and proliferation. (*k*) A green agent (S/G2/M phase) at lattice site *L* will successfully divide and transition if the randomly selected neighbouring site is vacant.

To simulate cell migration, agents undergo a nearest neighbour random walk at rates *M*_*r*_, *M*_*y*_, *M*_*g*_ per hour for red, yellow and green agents, respectively (figure 2*a*–*f*). Potential movement events involve randomly selecting the target site from the set of six nearest-neighbouring lattice sites, with the movement event being successful only if the target site is vacant. In this way, crowding effects are simply accommodated. To simulate transitions through the cell cycle, red agents are allowed to transition into yellow agents at rate *R*_*r*_ per hour (figure 2*h*,*i*), yellow agents to green agents at rate *R*_*y*_ per hour (figure 2*i*,*j*) and green agents into two red daughter agents at rate *R*_*g*_ per hour (figure 2*j*,*k*). While we assume that the red-to-yellow and yellow-to-green transitions are unaffected by crowding, we model crowding effects for the green-to-red transition by aborting transitions where the additional red daughter agent would be placed onto an occupied lattice site. By prohibiting multiple agents from occupying the same lattice site, we are able to realistically incorporate crowding effects [23,24].

The Simpson *et al.* [7] model is dependent on the initial geometry, boundary conditions, the lattice spacing Δ, and the cell cycle transition and motility rates. Since we have reasonable estimates for Δ [21] and we calibrate the initial geometry and boundary conditions to the experimental data, our study is concerned with estimating the unknown cell cycle transition and motility parameters. In a Bayesian setting, the unknown model parameters, *θ* = (*R*_*r*_, *R*_*y*_, *R*_*g*_, *M*_*r*_, *M*_*y*_, *M*_*g*_), and the uncertainty around them can be quantified by the posterior distribution, which is dependent on the likelihood and the prior distribution. However, while the Markov process model can capture the stochastic nature of cell proliferation and migration, when the dimension of the generator matrix (a matrix of rate parameters which describe the rate of transitioning between states) is too high the likelihood function consequently becomes intractable due to the computational cost of computing the matrix exponential (see [25–28]). Since conventional Bayesian approaches to parameter estimation are no longer feasible, we are motivated to use likelihood-free methods.

### 3.2. Approximate Bayesian computation

Using a Bayesian framework, the uncertainty about the unknown parameter *θ* with respect to the data *y* can be quantified by sampling from the posterior distribution *π*(*θ*|*y*) ∝ *π*(*y*|*θ*)*π*(*θ*), where *π*(*y*|*θ*) is the likelihood function and *π*(*θ*) is the prior. However, the likelihood function for sufficiently complex models becomes intractable (see examples in biology [12,24,29], in ecology [30,31] and in cosmology [32]). Rather than reverting to simpler models with tractable likelihoods, these types of problems can be instead analysed using likelihood-free methods that avoid evaluating the likelihood function.

One popular likelihood-free approach is ABC [33]. ABC involves simulating data from the model *x* ∼ *π*(· |*θ*) instead of evaluating the intractable likelihood; accepting configurations of *θ* which produce simulated data *x* that is close to the observed data *y*. It can be impractical to compare the full data sets of *x* and *y*, so ABC often relies on reducing the full data sets to summary statistics by some summarizing function *S*(·), where the summary statistics for *x* and *y* are denoted *S*_*x*_ = *S*(*x*) and *S*_*y*_ = *S*(*y*), respectively. Provided the summary statistics are highly informative about the model parameters, then *π*(*θ*|*y*) ≈ *π*(*θ*|*S*_*y*_) is a good approximation or exact if sufficient statistics are used [34]. However, the latter are usually difficult to attain in practice and so this study focuses on the use of summary statistics. In effect, ABC samples from the approximate posterior:

3.2 $$\pi_{\epsilon }(\theta |S_{y})\propto \pi (\theta )\int_{x}K_{\epsilon }(\rho (S_{y},S_{x}))\pi (x|\theta )\;dx,$$

where *ρ*(*S*_*y*_, *S*_*x*_) is the discrepancy function which measures the difference between the two data sets and $K_{\epsilon }(\cdot )$ is the kernel weighting function which weighs *ρ*(*S*_*y*_, *S*_*x*_) conditional on the tolerance *ε*. A common choice for the discrepancy function is the Euclidean distance, $\rho (S_{y},S_{x})={∥S_{y}-S_{x}∥}_{2}$, and for the kernel weighting function is the indicator function, 1(⋅), which is equal to one if *ρ*(*S*_*y*_, *S*_*x*_) ≤ *ε* and is zero otherwise. The approximate posterior in equation (3.2) converges to the posterior conditional on the observed summary (often referred to as the partial posterior) in the limit as *ε* → 0 [35].

To sample from the approximate posterior, commonly ABC-rejection [36,37], MCMC-ABC [38], or sequential Monte Carlo ABC (SMC-ABC) (e.g. [39–41]) algorithms are used. ABC-rejection samples particles from the prior distribution and accepts particles with a discrepancy measure *ρ*(*S*_*y*_, *S*_*x*_) less than the desired tolerance *ε*. In cases when the prior distribution is relatively diffuse compared to the posterior density (such as our application), lower acceptance rates are common because particles are predominantly sampled in regions of low posterior density [39]. To increase efficiency, one could instead use MCMC-ABC which constructs a Markov chain with a stationary distribution identical to the approximate posterior by proposing particles from a carefully-tuned proposal distribution, *θ*^*i*^ ∼ *q*(· |*θ*^*i*−1^), and accepting those with probability

3.3 $$p_{\mathrm{acc}}=\min\left(1,\frac{\pi (\theta^{i})q(\theta^{i-1}|\theta^{i})K_{\epsilon }(\rho (S_{y},S_{x}^{i}))}{\pi (\theta^{i-1})q(\theta^{i}|\theta^{i-1})K_{\epsilon }(\rho (S_{y},S_{x}^{i-1}))}\right),$$

which is based on the Metropolis–Hastings ratio [42,43]. While MCMC-ABC tends to be more computationally efficient compared to ABC-rejection [38], it is possible for the Markov chain to spend many iterations in areas of low posterior probability. In our application, we found MCMC-ABC to take a considerable effort to tune the proposal distribution while still being computationally cumbersome. However, SMC-ABC or more specifically the SMC-ABC replenishment algorithm [40] requires very little tuning comparatively and allows for simulations to be performed in parallel to increase computational efficiency.

The SMC-ABC replenishment algorithm traverses a set of distributions defined by *T* non-increasing tolerance levels *ε*_1_ ≥ · · · ≥ *ε*_*T*_ to sample from the approximate posterior:

$$\pi_{\epsilon_{t}}(\theta |S_{y})\propto \pi (\theta )\int_{x}1({∥S_{y}-S_{x}∥}_{2}\;\leq \epsilon_{t})\pi (x|\theta )\;dx,\mathrm{for}\;t=1,\ldots ,T,$$

where the first target distribution is constructed by sampling from the prior distribution to attain a collection of parameter values (called particles) and their discrepancies, ${\left\{\theta^{i},\rho^{i}\right\}}_{i=1}^{N}$. The first tolerance threshold, *ε*_1_, is set as the maximum of the set of discrepancies. Thereafter, to propagate particles through the sequence of target distributions, particles are first sorted in ascending order by their discrepancy and the new tolerance is set as $\epsilon_{t}=\rho^{N-N_{\alpha }}$ where $N_{\alpha }=\lfloor \alpha N\rfloor$, *α* is the proportion of particles discarded and ⌊⋅⌋ is the floor function. Particles, ${\left\{\theta^{i}\right\}}_{i=N-N_{\alpha }+1}^{N}$, which do not satisfy the new tolerance are discarded and resampled, with replacement from the remaining particles to replenish the population. To prevent sample degeneracy (too many duplicated particles), resampled particles are then perturbed according to an MCMC kernel *R*_*t*_ times with an invariant distribution given by the current approximate posterior $\pi_{\epsilon_{t}}(\theta |S_{y})$. In each of the *R*_*t*_ iterations, the proposed particles are drawn from an automatically tuned proposal distribution *θ*^*i*^ ∼ *q*( · |*θ*^*i*−1^) and accepted with probability $p_{\mathrm{acc}}^{i,j}$ (equation (3.3)) where *i* denotes the *i*th particle and *j* the *j*th MCMC iteration. To ensure sample diversity, *R*_*t*_ can be dynamically set based on the overall MCMC acceptance rate, $\bar{\;p_{\mathrm{acc}}}=1/(N_{\alpha }\times R_{t})\sum_{i=1}^{N_{\alpha }}$ $\sum_{\;j=1}^{R_{t}}p_{\mathrm{acc}}^{i,j}$, such that there is a 1 − *c* chance that all particles are moved at least once and is given by

$$R_{t}=\left\lceil \frac{\log(c)}{\log(1-\bar{\;p_{\mathrm{acc}}})}\right\rceil ,$$

where the ceiling function ⌈⋅⌉ is used to be conservative and an estimate for $\bar{\;p_{\mathrm{acc}}}$ is calculated from *R*_*t*−1_/2 pilot MCMC iterations. A popular choice for the proposal distribution is the multivariate normal distribution, $q(\theta^{i}|\theta^{i-1})=N(\theta^{i};\theta^{i-1},\Sigma )$, where the covariance matrix Σ is the tuning parameter. To create a more efficient proposal distribution, we can adaptively tune Σ by computing the empirical covariance matrix of the ${\left\{\theta \right\}}_{i=1}^{N-N_{\alpha }}$ particles which are already distributed according to the current target distribution. In this application, we do not worry about scaling the covariance matrix as there are only six parameters. The algorithm finally stops once the overall MCMC acceptance rate, $\bar{\;p_{\mathrm{acc}}}$, is unreasonably low (less than or equal to 1%) or the desired tolerance threshold is reached. For the two tuning parameters, Drovandi & Pettitt [40] suggest setting *α* = 0.5 and *c* = 0.01. The SMC-ABC replenishment algorithm is presented in algorithm 1 and is hereafter referred to as SMC-ABC.

A crucial limitation of ABC methods is the curse of dimensionality, where despite the addition of more data, the approximation to the posterior can become distorted as a result of the discrepancy between observed data and simulated data *ρ*(*S*_*y*_, *S*_*x*_) naturally increasing with the dimension [35]. In applications where increasing the dimension of the summary statistics cannot be avoided, the discrepancy between observed and summary statistics can be accounted for, at least approximately, with regression adjustment [34,35]. Regression adjustment involves explicitly modelling the parameters against the discrepancy between observed and simulated data. Assume for the moment that *θ* is a scalar parameter. Consider the following regression model

$$\theta^{i}=\beta_{0}+{\left(S_{x}^{i}-S_{y}\right)}^{⊤}\beta +\varepsilon^{i},$$

where *i* = 1, …, *N* is the parameter sample index, *β* is the regression coefficients, *β*_0_ is the intercept and ɛ^*i*^ is the error term. Estimates for *β*_0_ and *β* can be computed by minimizing the weighted least squares criterion $\sum_{i=1}^{N}w^{i}{\left(\theta^{i}-\beta_{0}-{\left(S_{x}^{i}-S_{y}\right)}^{⊤}\beta \right)}^{2}$. Here, we choose to use the popular Epanechnikov weighting function [44], defined as $w^{i}=0.75(1-{\left(\rho^{i}/\max({\left\{\rho^{i}\right\}}_{i=1}^{N})\right)}^{2})$, but other weighting functions could also be used. Using the estimated regression coefficients $\hat{\beta }$, we then make the adjustment

$$\theta^{i^{*}}=\theta^{i}-{\left(S_{x}^{i}-S_{y}\right)}^{⊤}\hat{\beta }\;\text{for }i=1,\ldots ,N.$$

The adjusted sample ${\left\{\theta^{i^{*}}\right\}}_{i=1}^{N}$ can often give a more accurate approximation of the posterior. To ensure that the adjusted parameters remain within the support of the prior distribution (if bounded), Hamilton *et al.* [45] suggest transforming parameter values before applying the regression adjustment. We use a logit transformation, $\tilde{\theta }=\log((\theta -a)/(b-\theta ))$, where *a* and *b* are the respective lower and upper bounds of the prior. Given that we have a vector of parameters, we apply a regression adjustment to each component of the parameter vector separately.


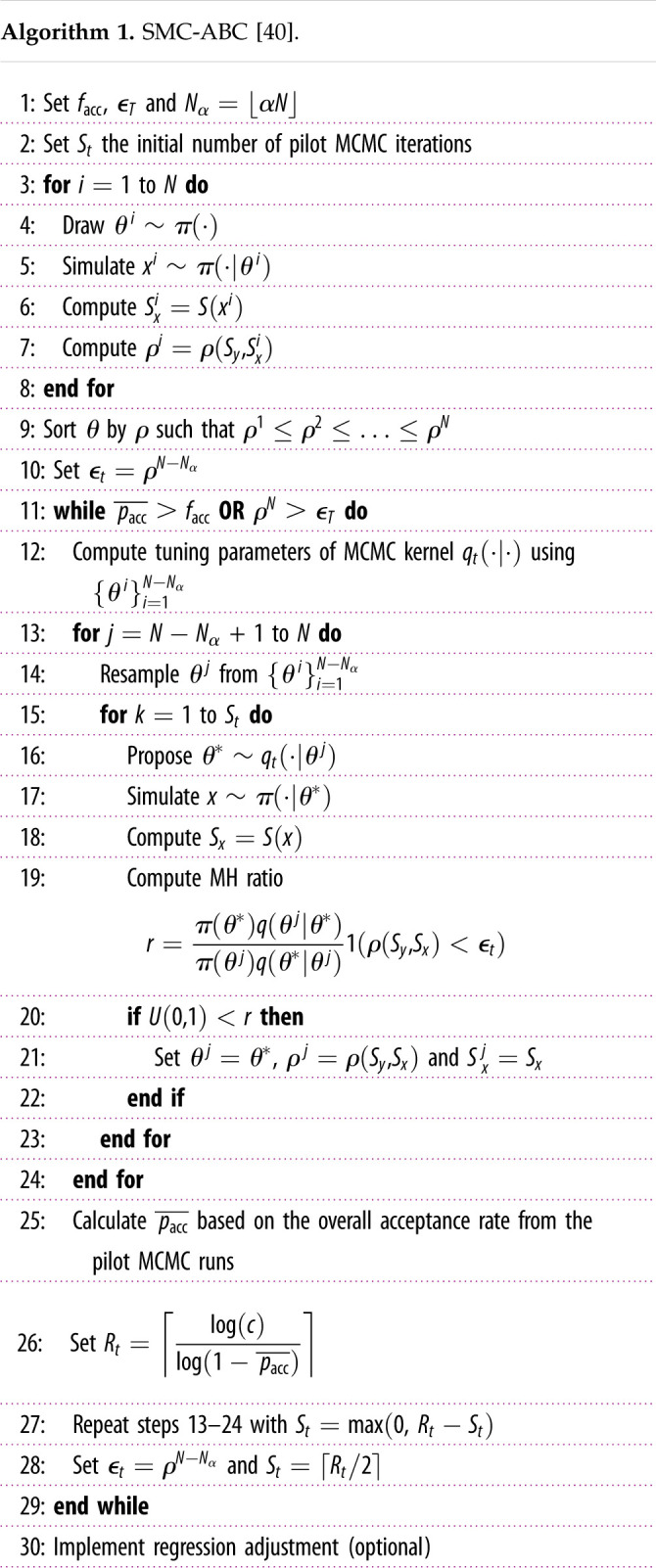


### 3.3. Prior knowledge

The cell cycle time, which is related to the doubling time, can be thought of as the summation of the time spent in each phase of the cell cycle. Estimates of the cell doubling time for melanoma cells range from 16 to 47 h [4,9]. Furthermore, Simpson *et al.* [9] estimate the average time 1205Lu FUCCI-transduced melanoma cells spend in the G1 to be between 8 and 30 h. This means that the transition from G1 to S/G2/M phase is approximately 1/30−1/8 h^−1^. Additionally, the duration spent in S/G2/M phases were reported as 8–17 h. This means that the transition from S/G2/M to G1 phase is approximately 1/17–1/8 h^−1^. Therefore, we propose that our prior information of the transition rates is uniform over the range 0–1 h^−1^ to be conservative.

Cell diffusivity, *D*, the measurement of motility rate for particles undergoing random diffusive migration, can be used to quantify the cell motility rate $M\;\in \;\{M_{r},M_{y},M_{g}\}$, by *D* = *M*Δ^2^/4 [46], where Δ is the cell diameter. Empirical evidence finds estimates for cell diffusivity to range from 0 to 3304 μm^2^ h^−1^ [13,14,21]. Furthermore, Simpson *et al.* [7] suggest the cell diffusivity to be approximately 400 μm^2^ h^−1^, so that the rates are approximately 4 h^−1^. We propose that the prior information of the motility rates to be uniform over the range 0−10 h^−1^; attributing the larger interval to the greater variation of cell diffusivity estimates in existing literature.

## 4. Results

For SMC-ABC, we generate samples from the approximate posteriors using *N* = 1000 particles. From preliminary trials, we found it more useful to use the overall MCMC acceptance rate as the stopping rule for the SMC-ABC algorithm and adopt the sensible choice for the final acceptance rate as $f_{\mathrm{acc}}=1\text{\%}$ and *ε*_*T*_ = 0 for the target tolerance.

### 4.1. Developing summary statistics and validation with synthetic data

The accuracy and precision of ABC methods in approximating the posterior distribution is sensitive to the quality of the summary statistics used [35]. We first trial and validate different summary statistics with multiple synthetic datasets such that the true parameter values are known. In this way, we are able to compare the performance of different summary statistics and determine which are the most effective. While trying to replicate the environment of the experimental data as close as possible, such as domain size, boundary conditions and initial number of cells, we do not calibrate the initial location of cells but rather randomly distribute the cells within a 200 μm by 1745.35 μm region on either side of the scratch. We attain the initial cell counts of red, yellow and green cells by using the procedure outlined in §4.2 (steps 1–4) and report them here to be 119, 35 and 121, respectively.

In our analysis of the simulation model, agents with relatively higher transition rates were found to correspond to lower population sizes, and vice versa. Therefore, we use the number of agents in each population (*N*_*r*_, *N*_*y*_, *N*_*g*_) at the end of the experiment as summary statistics that may be informative about the transition rates *R*_*r*_, *R*_*y*_, *R*_*g*_, respectively. We test the suitability of this summary statistic on four synthetic data sets produced by varying the transition rates amongst biologically plausible values and keeping the motility rates known and constant. Estimates in existing literature of cell transition rates are similar [4,9] and the efficiency of the simulation model is dependent on the number of agents in the system (higher transition rates increase the overall proliferation rate and the frequency of events). Therefore, we choose to keep the transition rates rather close to the estimates of Haass *et al.* [4], instead of varying them over the extents of the prior domain. Thus, the four parameter configurations we choose to generate the synthetic datasets are θ ∈{(0.04,0.17,0.08,4,4,4),(0.25,0.15,0.22,4,4,4),(0.12,0.07,0.03,4,4,4),(0.3,0.36,0.28,4,4,4)}. In §2 of the electronic supplementary material, we present the marginal posterior distributions produced and confirm the suitability of this summary statistic.

For the motility parameters, we explore and compare two sets of summary statistics, namely cell density and cell trajectory data. Of these two datasets, cell density data are desirable due to less manual effort needed to generate the data while cell trajectory data could offer more information but is more challenging to collect. For the cell density data, we first segment the imaged region at the end of the experiment (*t* = 48 h) directly down the centre of the image in the *y* direction and calculate the median position and interquartile ranges of the red, yellow and green agent populations in the *x*-direction for cells on the left and right sides. For the cell trajectory data, we average the distance of multiple cell trajectories through each cell phase until the cell returns to the initial phase or the simulation is terminated. We select cells to be tracked provided that the cell is initially in G1 (red) phase and the cell is located on the leading edge of the cell monolayer toward the gap in the scratch assay. The reasoning behind beginning tracking from the G1 phases is due to a short period of fluorescent negativity in between S/G2/M phases and G1 phase [4] which makes tracking between these phases difficult. Therefore, by starting from the G1 phase, we avoid having to track between these two phases. Furthermore, cells were identified as being on the leading edge if their path towards the middle of the scratch was unhindered. The process of manually tracking cell trajectories can be time consuming. Thus, we are interested in finding the minimum number of cells to track such that sufficient information is acquired. In §2 of the electronic supplementary material, we draw samples from the posterior distribution using 10, 20, 30, 40 and 50 cell trajectories with four synthetic data sets generated from θ ∈{(0.04,0.17,0.08,4,4,4),(0.04,0.17,0.08,2,5,8), (0.04,0.17, 0.08,8,2,5),(0.04,0.17,0.08,8,2,5),(0.04,0.17,0.08,5,8,2)}. Our analysis finds diminishing returns of parameter precision as the number of cell trajectories increases. We find that using 20 cell trajectories achieves a good balance between precision and the number of cell trajectories used. Using the same four synthetic datasets, we also attempt to draw samples from the posterior distribution using cell density data with the cell transition rates held constant in §2 of the electronic supplementary material. However, under these settings, we found the motility parameters to be non-identifiable.

**Figure 3.**
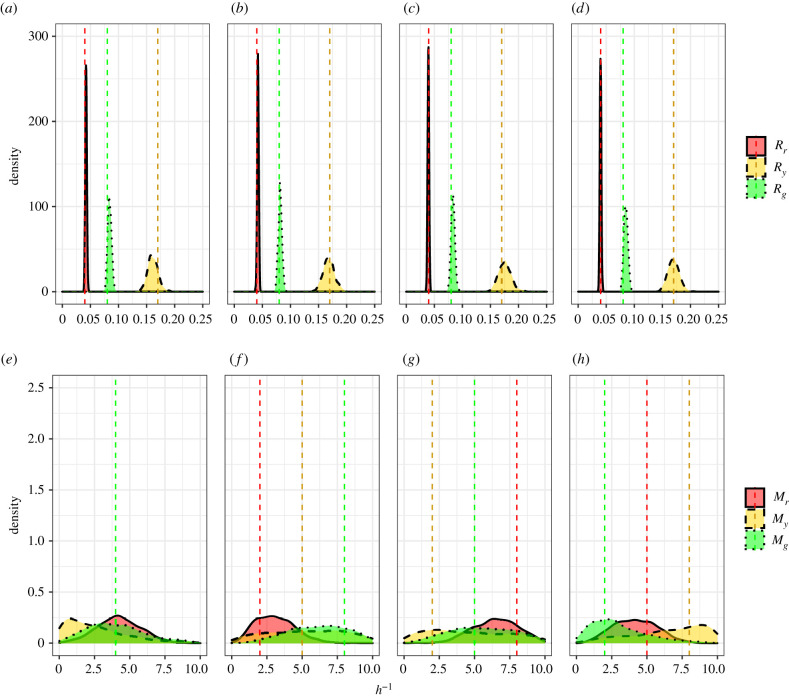
Estimating cell cycle transition and cell motility parameters, *θ* = (*R*_*r*_, *R*_*y*_, *R*_*g*_, *M*_*r*_, *M*_*y*_, *M*_*g*_), with the number of cells in each phase at *t* = 48 h and cell density data as summary statistics across several synthetic data sets. Synthetic datasets were produced from simulations with true parameter values indicated by dashed vertical lines (note that in (*e*) the lines overlap). (*a*,*e*) Estimated marginal posteriors produced with *θ* = (0.04, 0.17, 0.08, 4, 4, 4). (*b*,*f*) Estimated marginal posteriors produced with *θ* = (0.04, 0.17, 0.08, 2, 5, 8). (*c*,*g*) Estimated marginal posteriors produced with *θ* = (0.04, 0.17, 0.08, 8, 2, 5). (*d*,*h*) Estimated marginal posteriors produced with *θ* = (0.04, 0.17, 0.08, 5, 8, 2).

**Figure 4.**
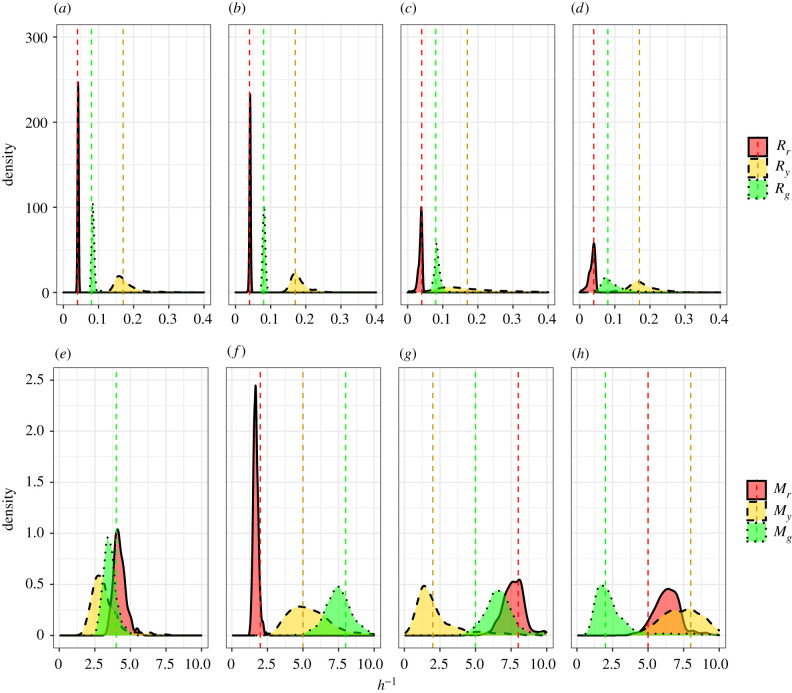
Estimating cell cycle transition and cell motility parameters, *θ* = (*R*_*r*_, *R*_*y*_, *R*_*g*_, *M*_*r*_, *M*_*y*_, *M*_*g*_), with the number of cells in each phase at *t* = 48 h and cell tracking data as summary statistics across several synthetic datasets. Synthetic datasets were produced from simulations with true parameter values indicated by dashed vertical lines (note that in (*e*) the lines overlap). (*a*,*e*) Estimated marginal posteriors produced with *θ* = (0.04, 0.17, 0.08, 4, 4, 4). (*b*,*f*) Estimated marginal posteriors produced with *θ* = (0.04, 0.17, 0.08, 2, 5, 8). (*c*,*g*) Estimated marginal posteriors produced with *θ* = (0.04, 0.17, 0.08, 8, 2, 5). (*d*,*h*) Estimated marginal posteriors produced with *θ* = (0.04, 0.17, 0.08, 5, 8, 2).

We now combine the summary statistics formulated to estimate the cell cycle transition and motility rates together with four synthetic data sets. Due to the similarity in estimates for cell cycle transition rates in existing literature (see [4,9]), we adopt estimates for the cell cycle transition rates from Haass *et al.* [4] for all four parameter configurations. Since estimates for motility rates have been reported to vary by two orders of magnitude (see [13,14,21]), we choose to vary the motility rates over the range of the prior for the four parameter configurations. That is, we generate four synthetic data sets with *θ* ∈ {(0.04, 0.17, 0.08, 4, 4, 4), (0.04, 0.17, 0.08, 2, 5, 8), (0.04, 0.17, 0.08, 8, 2, 5), (0.04, 0.17, 0.08, 5, 8, 2)}. In figure 3, we present the marginal posterior distributions produced when using the number of cells in each subpopulation and cell density data as summary statistics. In figure 4, we present the marginal posterior distributions when using cell trajectory data in place of cell density data. Again, we see that the motility parameters are practically non-identifiable when cell density data are used while both cell cycle transition and motility parameters are practically identifiable when cell trajectory data are included. Furthermore, it is clear from the concentration of the marginal posterior distributions around the true parameter values (dashed line) in figure 4 that the cell count and cell trajectory data are highly informative about the transition and motility parameters, respectively. We note that the precision of these distributions is greater for the cell cycle transition parameters than the motility parameters. Importantly, these results show for the first time that practical parameter inference on both transition and motility parameters of a FUCCI scratch assay experiment using Bayesian inference techniques is possible. These results justify the choice of the Markov process model compared to simpler continuum models which do not give insight into cell trajectory data (see [9]).

### 4.2. Image analysis of experimental data

**Figure 5.**
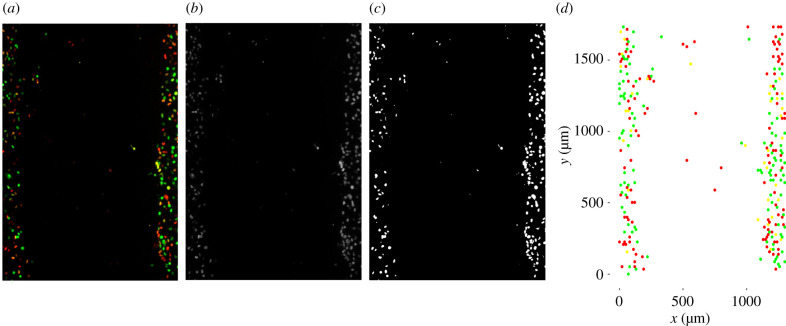
*ImageJ* procedure. (*a*) Original image loaded (WM983C FUCCI-transduced melanoma cells). (*b*) Image after compression to 8-bit. (*c*) Image after converting to black and white and watershedding. (*d*) Simulation initial geometry recovered from data processing of WM983C FUCCI-transduced melanoma cells in *ImageJ* and *R*.

We analyse the experimental images using *ImageJ* [47] to record the Cartesian coordinates of cells. Of primary interest is processing the initial frame such that we can replicate the experimental settings as accurately as possible in the simulation but we also repeat this procedure for the final frame to retrieve the final cell counts and cell density data, which we use as summary statistics. The process is as follows:

Step 1: *Read in image:* File > Open > *select image* (figure 5*a*).
Step 2: *Convert image to 8-bit:* Image > Type > 8 − *bit* (figure 5*b*).
Step 3: *Identify cell edges:* Convert the image to black and white (Process>Binary>convert to mask) and then distinguish conjoined cells (Process > Binary > Watershed) (figure 5*c*).
Step 4: *Compute Cartesian coordinates:* Analyse > Analyse particles…> OK.

A limitation of using the watershed tool is that we must convert the image to black and white. In doing so, we lose the cell phase identity associated with the cell coordinates recovered from *ImageJ*. To overcome this, we use *R* [48] to retrieve the RGB decimal colour code and Cartesian coordinates of pixels. Matching pixel coordinates recovered from *R* and the coordinates of the centroid of the cells recovered in *ImageJ*, we create a data set of cell coordinates and their associated RGB decimal codes. To classify the RGB coordinates into one of the three cell cycle phases we use the conditions outlined in table 1.

**Table 1.** Cell phase classification rule using RGB decimal codes.

state	RGB decimal code	
	red	green
G1	>100	≤100
eS	>100	>100
S/G2/M	≤100	>100

**Figure 6.**
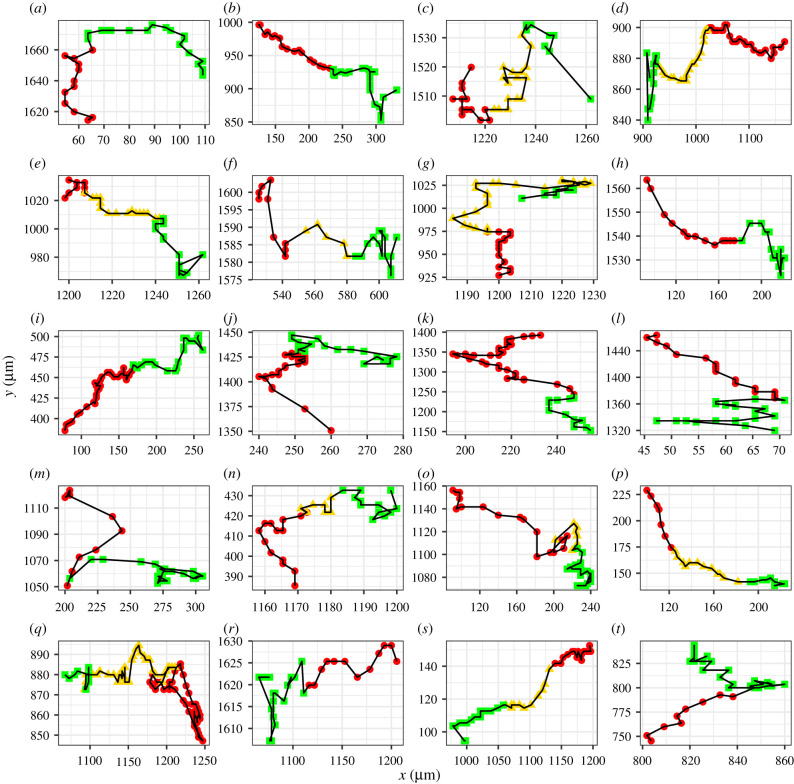
Trajectories of WM983C FUCCI-transduced melanoma cells where each box (*a*–*t*) corresponds to one of the 20 cell trajectories. Tracking begins in red phase (red circles) then progresses through the yellow phase (yellow triangles) and is terminated at end of green phase (green squares).

To extract summary statistics from the experimental data, we repeat the image processing procedure previously outlined above with the final frame (*t* = 48 h) and extract the final cell counts and cell density data. Additionally, we extract cell trajectory data by processing the entire sequence of still images in *ImageJ* with the ‘Multi-point’ tool to manually track cell coordinates between frames. We use a similar process as before to identify cell phases in these summary statistics using *R* and present them in table 2 and the cell trajectory data in figure 6.

**Table 2.** Observed summary statistics of WM983C FUCCI-transduced melanoma cells

summary statistic	description	value
*S*_1_	number of red cells at 48 hours	566 cells
*S*_2_	number of yellow cells at 48 hours	111 cells
*S*_3_	number of green cells at 48 hours	166 cells
*S*_4_	average distance travelled through red phase by 20 cells	105 μm
*S*_5_	average distance travelled through yellow phase by 20 cells	40 μm
*S*_6_	average distance travelled through green phase by 20 cells	100 μm
*S*_7_	median position of red cells on the left and right side	(155, 1170) μm
*S*_8_	median position of yellow cells on the left and right side	(158, 1189) μm
*S*_9_	median position of green cells on the left and right side	(177, 1129) μm
*S*_10_	interquartile range of the red cells position on the left and right side	(196, 197) μm
*S*_11_	interquartile range of the yellow cells position on the left and right side	(164, 144) μm
*S*_12_	interquartile range of the green cells position on the left and right side	(213, 207) μm

Finally, we calibrate the hexagonal lattice used in the simulation model with the dataset of Cartesian coordinates recovered previously by rearranging equation (3.1) to find their associated lattice row and column indices denoted

$$(i,j)=\left(\left\lfloor \frac{2x}{\sqrt{3}\Delta }+1\right\rceil ,\left\lfloor \frac{y}{\Delta }\right\rceil \right),$$

where ⌊⋅⌉ rounds to the nearest integer. We treat the rare instances (<1%) where multiple coordinates are mapped to the same lattice space as duplicated values and omit them rather than place them on the next closest lattice site. The result from this translation of data is presented in figure 5*d*. We repeat this process for the initial frame of the cell trajectory data to identify the starting position. However, due to manually tracking cell trajectories, often the coordinate retrieved was not centred on the cell which in some cases caused the starting position to be mapped to an unoccupied lattice site. We intervene prior to transforming the starting position and adjust the coordinate values to the closest occupied lattice site which is chosen such that the radial distance between the coordinate and the lattice site is minimized.

### 4.3. Estimating model parameters with experimental data

**Figure 7.**
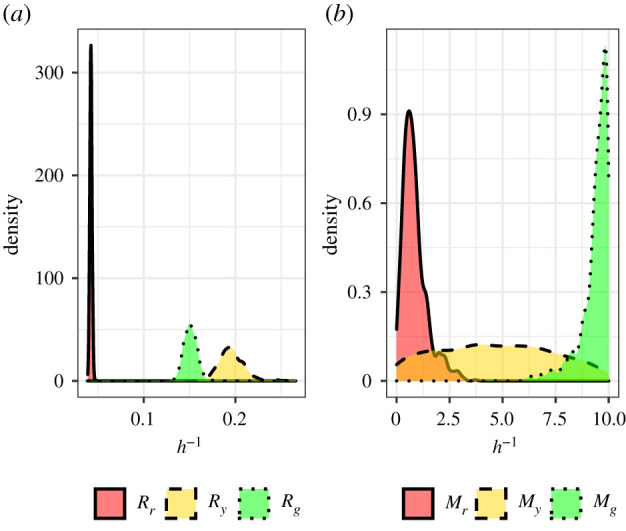
Marginal posterior distributions using number of cells in each subpopulation and cell density data. (*a*) Marginal posterior distributions for transition rates of WM983C FUCCI-transduced melanoma cells. (*b*) Marginal posterior distributions for motility rates of WM983C FUCCI-transduced melanoma cells.

After calibrating the simulation to the experimental data of WM983C FUCCI-transduced melanoma cells, we first attempt to sample from the posterior distribution using the number of cells in each subpopulation and cell density data (summary statistics *S*_1_ to *S*_3_ and *S*_7_ to *S*_12_ in table 2, respectively) and present the samples from the posterior distribution in figure 7. Consistent with results found in §4.1 and Simpson *et al.* [9], estimates for the motility rates are practically non-identifiable when cell density data are used.

**Figure 8.**
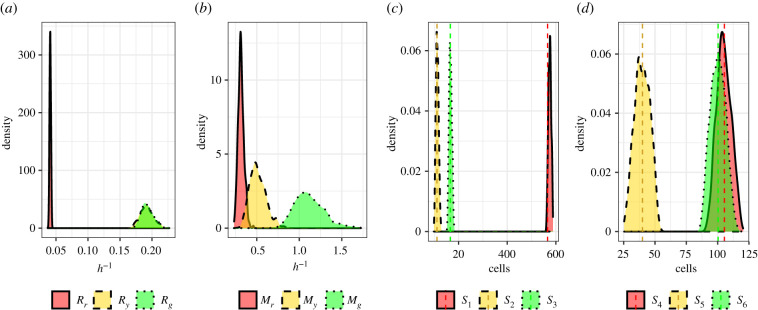
Marginal posterior distributions using number of cells in each subpopulation and cell trajectory data. (*a*) Marginal posterior distributions for transition rates of WM983C FUCCI-transduced melanoma cells. (*b*) Marginal posterior distributions for motility rates of WM983C FUCCI-transduced melanoma cells. (*c*) Distribution of simulated summary statistics (informative of transition rates) compared to observed summary statistics (dashed line). (*d*) Distribution of simulated summary statistics (informative of motility rates) compared to observed summary statistics (dashed line).

Next, we attempt to sample from the posterior distribution using the number of cells in each subpopulation and cell trajectory data (summary statistics *S*_1_ to *S*_3_ and *S*_4_ to *S*_6_ in table 2, respectively). We present the marginal posterior distributions produced in figures 8*a*,*b* along with the mean, standard deviation, (2.5%, 50%, 97.5%) quantiles, and the coefficient of variation (CV) in table 3. The practical identifiability in the transition and motility parameters clearly shows the benefits of using cell tracking data as the distributions are unimodal and concentrated. We estimate the cell cycle transition rates to be between 0.0411−0.193 h^−1^ which is consistent with estimates in existing literature [4,9]. Our estimates for cell motility were found to range between 0.316−1.12 h^−1^ which corresponds to estimates of cell diffusivity between 31.6 and 112 μm^2^ h^−1^ which is reasonable considering the degree of uncertainty in existing estimates which can vary between 0 and 3304 μm^2^ h^−1^ [13,14,21]. The precision in parameter estimates can be quantified by the CV which is a standard measure for the dispersion of data around the mean. Using the CV, the dispersion in the transition rates range from 2.65 to 5.31% and the motility rates range from 10.9 to 18.4%. To validate the parameter estimates recovered, we also present the posterior predictive distributions for the summary statistics retained from each parameter value in the posterior in figures 8*c*,*d*. These distributions are formed by plotting the distribution of simulated summary statistics produced from the posterior samples and is compared to the observed summary statistics (dashed line). These results suggest that the Markov process model developed by Simpson *et al.* [7] is promising as it is able to recover the observed summary statistics of the experimental data. However, further model validation should be considered in future research to determine if the simulated cell trajectories produce similar paths to those which were observed.

**Table 3.** Posterior summaries (three significant figures): mean, standard deviation, (2.5%, 50%, 97.5%) quantiles and the coefficient of variance (CV).

parameter	mean	s.d.	(2.5%, 50%, 97.5%)	CV (%)
*R*_*r*_	0.0411	0.00109	(0.039, 0.0411, 0.0432)	2.65
*R*_*y*_	0.192	0.0102	(0.173, 0.191, 0.214)	5.31
*R*_*g*_	0.193	0.00957	(0.177, 0.192, 0.213)	4.96
*M*_*r*_	0.316	0.0343	(0.256, 0.313, 0.385)	10.9
*M*_*y*_	0.514	0.0945	(0.353, 0.502, 0.725)	18.4
*M*_*g*_	1.12	0.169	(0.836, 1.10, 1.48)	15.1

## 5. Discussion

In this study, we calibrate the 2D hexagonal-lattice random walk model developed by Simpson *et al.* [7] to scratch assay data where the cell cycle is revealed in real time using FUCCI technology. While this model is well suited to describing the stochastic nature of cell proliferation and migration, the likelihood function consequently becomes intractable. This makes conventional Bayesian approaches to parameter inference infeasible. We resort to using the class of Bayesian methods known as ABC which bypass evaluating the likelihood function. After evaluating the appropriateness of different ABC algorithms in §3.2 we find the SMC-ABC replenishment algorithm developed by Drovandi & Pettitt [40] to be suitable.

In this study, we work with uniform prior distributions. This may be considered as a reasonably vague prior, but it can also be interpreted as providing more support to larger rate parameters when the prior ranges over several orders of magnitude. To discourage larger rate parameter values, other priors could be considered, such as Jeffry’s prior over all positive reals. If a proper prior (i.e. integrates to unity) is desired, the Jeffrey’s prior could be truncated or an exponential prior used instead. We leave such extensive prior sensitivity analysis and performance for future research.

In §3.1, we previously discussed the intractability of the likelihood function. Although, we did not discuss particle filtering methods to construct a continuous time likelihood function which can be considerably more tractable than those based off discrete data. However, given that the model is highly stochastic, very different cell trajectories can be produced with the same parameter values. This would make filtering approaches difficult to apply. Instead, it is more efficient to match summary statistics of the cell trajectories (here we consider the average time spent in each phase). Additionally, pseudo-marginal MCMC [49] could be used to sample from the exact posterior distribution if an unbiased likelihood estimator (based on the full dataset) with a small enough variance can be constructed. Unfortunately, due to the complexity of the model and the need to summarize the data, it does not seem feasible to construct such a likelihood estimator here. Therefore, the nature of the modelling approach and the use of summary statistics naturally lends itself to using ABC methods.

The accuracy of ABC methods in approximating the posterior distribution is sensitive to the quality of the summary statistics used [35]. We trial various summary statistics with multiple synthetic data sets to determine which summary statistics are the most informative. We find using the number of cells in each cell cycle phase at the end of the experiment to be highly informative about the cell cycle transition rates. We trial and compare two sets of summary statistics for the motility parameters: the median position and interquartile range of the cells in the *x*-direction on the left and right side of the scratch assay (which we refer to as cell density data); and the average distance travelled through each cell phase by 20 individual cells (which we refer to as cell trajectory data). Using these two sets of summary statistics in conjunction with the cell count data, we attempt to draw samples from the posterior distribution using the SMC-ABC replenishment algorithm with multiple biologically plausible synthetic data sets. We find that when using cell trajectory data as summary statistics the parameters are practically identifiable; however this is not the case when cell density data are used. Importantly, this is the first time practical parameter identifiability for both cell cycle transition and motility has been successfully conducted with fluorescent cell cycle labelling scratch assay experiments.

In this study, we summarize cell trajectory data by taking the average distance travelled in each phase across 20 cell trajectories. However, additional features of cell trajectories could also be considered (for example, the variance). Although, the addition of more summary statistics may increase ABC error due to the increased dimensionality of the summary statistic despite our efforts to treat this with regression adjustment. An additional method which could be used is semi-automatic ABC [50] which constructs a set of summary statistics with the same dimension as the parameter space by modelling the importance of the initial set of summary statistics. However, exploration of these additional summary statistics and the the effects on the precision of the posterior distribution are left for future research.

We extend on the work of previous studies [7,9] by calibrating our model to real data and performing Bayesian inference. Using experimental data of WM983C FUCCI-transduced melanoma cells, we estimate the approximate posterior using the SMC-ABC algorithm with our cell cycle transition rate summary statistics and our two sets of motility summary statistics. Under the experimental setting, our results again find the estimates for the motility parameters to be practically non-identifiable when cell density data are used but practically identifiable when cell trajectory data are used. These results are consistent with Simpson *et al.* [9] and justify the motivation to use a stochastic model capable of generating multiple data types. When using the number of cells in each subpopulation and cell trajectory data, we find estimates for the average cell cycle transition rates to range between 0.0411−0.193 h^−1^ and estimates for average cell motility to range between 0.316−1.12 h^−1^. Interestingly, we find that the motility rates appear to depend upon the cell cycle phase and for these data the motility of cells in S/G2/M phase is higher than the motility rate in the in G1 or eS phase. We quantify the precision of these estimates through the CV which is a standard measure of dispersion about the mean. We find the CV to be suitably small for all parameters as it ranges from 2.65–5.31% and 10.9−18.4% for the transition and motility marginal posteriors, respectively. To validate our results, we also draw samples from the posterior predictive distribution to determine whether the simulated data sets recovered accurately reflect the observed datasets. These results confirm that the model and summary statistics are recovering the underlying mechanisms present in the experiment.

Now that the recovery of precise parameter estimates from a fluorescent cell cycle labelling model has been demonstrated, further models can be built which are more biologically realistic. For instance, the Markov process model we used in this study describes a discrete exclusion based random walk on a 2D hexagonal lattice. However, a more biologically realistic and meaningful model would incorporate a three-dimensional (3D) environment (e.g. [51]). By constraining our model to a 2D hexagonal lattice, we ultimately omit realistically modelling: the spatial supply of oxygen, nutrients and drugs; the orientation in 3D space; and interactions with the extracellular matrix [19,52]. Although, increasing model complexity tends to require additional parameters in the model which in some applications may render ABC methods ill suited to inference due to their poorer performance in higher dimensions [50]. Such modelling and inference implications would need to be considered in future work. Nevertheless, we demonstrate that the 2D stochastic model developed by Simpson *et al.* [7] is able to recover key features of the experimental data set we examined and can be used to provide a quick and inexpensive alternative to *in vitro* experiments.

Finally, to bypass evaluating the likelihood function we resort to using ABC techniques. However, ABC requires many model simulations, which can be computationally expensive if the simulation model is relatively inefficient. In our application, the computation time for the model is largely dependent on the value of the transition and motility parameters, where larger values will require more computation. We compute the computational cost of 1000 simulations with parameter configurations drawn from the prior distribution and report the computational cost of the model as a 95% empirical confidence interval that ranges between 1.08 and 57.33 s per simulation. Using an Intel(R) Xeon(R) Gold 6140 CPU at 2.3 GHz and paralysing over the 16 cores results in the total computation time of the SMC-ABC algorithm taking approximately 23 h to run when using cell count and cell trajectory data and 16 h when using cell count and cell density data. We find these computation times to be reasonable but future work may need to consider more computationally efficient modelling and/or statistical methods, particularly if more summary statistics are to be considered.

## Data accessibility

Rcpp and MATLAB implementations of the simulation model and SMC-ABC algorithm along with the data used in this study are available at https://github.com/michaelcarr-stats/FUCCI.
